# The impact of disseminating the whole-community project '10,000 Steps': a RE-AIM analysis

**DOI:** 10.1186/1471-2458-11-3

**Published:** 2011-01-04

**Authors:** Ragnar Van Acker, Ilse De Bourdeaudhuij, Katrien De Cocker, Lisa M Klesges, Greet Cardon

**Affiliations:** 1 Department of Movement and Sports Sciences, Ghent University, Watersportlaan 2, 9000 Gent, Belgium; 2 School of Public Health, University of Memphis, Scates Hall 205, Memphis, USA

## Abstract

**Background:**

There are insufficient research reports on the wide-scale dissemination of effective whole-community physical activity (PA) programs. The purpose of this paper is to evaluate the impact of the wide-scale dissemination of '10,000 Steps', using the RE-AIM framework.

**Methods:**

Dissemination efforts targeted a large region of Belgium and were concentrated on media strategies and peer networks of specific professional organizations, such as local health promotion services. Heads of department of 69 organizations received an on-line survey to assess project awareness, adoption, implementation and intended continuation of '10,000 Steps'. On the individual level, 755 citizens living in the work area of the organizations were interviewed for project awareness and PA levels. Measures were structured according to the RE-AIM dimensions (reach, effectiveness, adoption, implementation, maintenance). Independent sample *t *and chi-square tests were used to compare groups for representativeness at the organizational and individual level, and for individual PA differences.

**Results:**

Of all organizations, 90% was aware of '10,000 Steps' (effectiveness - organizational level) and 36% adopted the project (adoption). The global implementation score was 52%. One third intended to continue the project in the future (maintenance) and 48% was still undecided. On the individual level, 35% of citizens were aware of '10,000 Steps' (reach). They reported significantly higher leisure-time PA levels than those not aware of '10,000 Steps' (256 ± 237 and 207 ± 216 min/week, respectively; *t *= -2.8; p < .005) (effectiveness - individual level). When considering representativeness, adoption of '10.000 Steps' was independent of most organizational characteristics, except for years of experience in PA promotion (7.6 ± 4.6 and 2.9 ± 5.9 years for project staff and non-project staff members, respectively; *t *= 2.79; *p *< 0.01). Project awareness in citizens was independent of all demographic characteristics.

**Conclusions:**

'10,000 Steps' shows potential for wide-scale dissemination but a supportive linkage system seems recommended to encourage adoption levels and high quality implementation.

## Background

Translating research findings into evidence-based public health practices that are widely disseminated and adopted has been identified as one of the biggest challenges facing contemporary health promotion [1,2]. Effective evidence-based physical activity [PA] intervention studies in adults are available [3,4]. However, information on the wide-scale dissemination of evidence-based PA interventions into practice is also needed [5]. This dearth of dissemination evidence [1], as well as the growing concerns among policy-makers, researchers, and practitioners about the gap between research and practice have led to an increasing interest in dissemination and implementation research [6,7].

To support translation of evidence-based PA interventions into practice, generalizability or external validity characteristics of basic and community-based PA interventions should be addressed [6,8]. However, systematic reviews document that generalizability characteristics are not consistently reported in research reports on PA interventions [9,10]. Reports of community-wide PA intervention lack vital external validity information, especially related to the uptake and delivery of programs by community practitioners, important for determining a programs' public health impact [9].

Acknowledging this practical need for dissemination of evidence-based PA programs, the Government of Flanders (Dutch speaking part of Belgium) provided funding in 2007 to study the dissemination and implementation of a whole-community project called '10,000 Steps' in the entire region of Flanders. The proto-type project of '10,000 Steps' was originally developed in Australia [11,12], and was tested in 2005-2006 for European communities in the Flemish city of Ghent (for full description see [13]). The '10,000 Steps Ghent' project included multiple community-based strategies to promote PA in the adult population and was guided by the socio-ecological model [14], designed to intervene at the personal (e.g. pedometer sale and loan), social (e.g. dissemination of information through all partner-associations), and environmental (e.g. walking circuits) levels [13]. An increase of 8% in the number of people reaching the "10,000 steps" standard was seen after a year, compared with no increase in the comparison community. Significant intervention effects were also found for self-reported minutes of walking, and moderate, work-related, and leisure-time PA. However, the wide-scale dissemination of '10,000 Steps' outside the pilot project area requires further study.

Therefore, this paper reports on the impact of the wide-scale dissemination of '10,000 Steps' to the entire region of Flanders. 'Dissemination' was considered as an active approach of spreading an evidence-based intervention (i.e. '10,000 Steps') to the target audience via determined channels using planned strategies [15]. Rogers' Diffusion of Innovations theory [6,7,16] was used to guide dissemination efforts and the RE-AIM framework to evaluate and analyze the impact of '10,000 Steps'[17]. The RE-AIM framework has previously been applied to evaluate dissemination efforts for PA programs [5]. It assesses the reach, effectiveness, adoption, implementation, and maintenance of a project in order to estimate its public health impact. Moreover, this framework is compatible with a socio-ecological model and applied public health interventions [17], making it a particularly suitable framework for the evaluation of '10,000 Steps'.

## Methods

### Development

This study tests a third phase of '10,000 Steps' adapted from the original '10,000 Steps Rockhampton' study from Australia [11,12] and the subsequent pilot study '10,000 Steps Ghent' [13]. Based on these two local intervention studies, intervention guidelines for '10,000 Steps' were formulated and disseminated in the present study in Flanders. These guidelines were addressed to potential adopters of '10,000 Steps' and several intervention components were recommended. Table 1 provides an overview of the intervention components that were recommended to potentially adopting organizations in the present study in Flanders compared to the implemented intervention components of '10,000 Steps Rockhampton' and '10,000 Steps Ghent'.

**Table 1:** Socio-ecological intervention components and dissemination strategies of '10,000 Steps' studies

	*10,000 Steps Rockhampton(2 year project)*	*10,000 Steps Ghent(1 year pilot)*	10,000 Steps in Flanders(present study)*
**Intrapersonal**	Sale (general practitioner (GP), health services) and loan (libraries and video shops) of pedometers	Sale (local town shop, health services) and loan (sport service) of pedometers	Sale and loan of pedometers in every municipality (local public services)
	Website of 10,000 Steps Rockhampton	Website of 10,000 Steps Ghent	Website updated from 10,000 Steps Ghent
**Interpersonal**	Promotion of PA by health professionals and print media	Promotion of PA and distribution of folders through GP's, dieticians, physical therapists and schools; posters in public places	Promotion of PA and distribution of folders and posters in public places
	Not specified	Not specified	Personalized contact with citizens (e.g. personalized letter, mail, or phone)
**Organizational**	Community events, specific projects for GPs, health services involvement and for workplaces	Community events, specific projects for workplaces and for groups of older people	Community events, projects for the entire population and all domains of active living (PA for transport, at work, for household and leisure time)
**Community**	Local mass media campaign	Local media campaign	Local mass media campaign in every municipality
	10,000 Steps a day - Every Step Counts	10,000 Steps a day - Every Step Counts, 30 minutes MVPA guideline	10,000 Steps a day - Every Step Counts, 30 MVPA minutes guideline
	Environmental: street signs, distribution of maps, promotion of dog walking	Environmental: street signs, walking circuits and billboards	Environmental: street signs, billboards...
**Policy**	Partnerships between local government and key members of community organizations, some with high-level experience in PA promotion	Partnerships between the local city and provincial government, health insurance companies, and the local health promotion service	Partnerships between the adopting organization and minimum one (other) local government service or two professional organizations
	Sale and loan of pedometers	Sale and loan of pedometers	Sale and loan of pedometers

**Strategies for dissemination among potential adopter(s)**	Local: Recruitment of community partners by researchers (micro grants) to form a local PA task force, GP training	Local: Recruitment of community partners by researchers to form a local steering committee	Regional: website, mailing of the project manual and pilot study results, group meetings, displays at conferences, e-articles

* The intervention components described for the present study in Flanders were recommended to potentially adopting organizations (guidelines), and therefore do not represent actual implementation.

The intervention components across all three phases of testing represent a socio-ecological approach to PA promotion in adults, with some small adaptations throughout the phases [18]. Main adaptations included less focus on delivery via general practitioners and dog walking, and a stronger focus on promoting public places in both '10,000 Steps Ghent' and the present study in Flanders. To emphasize the importance of the interpersonal dimension of PA promotion, wide ranging personal contact with citizens (e.g. personalized letter, mail, or phone) was included as an additional intervention guideline in the present study in Flanders. Furthermore, in '10,000 Steps Ghent' and the present study in Flanders the guideline of 30 minutes moderate to vigorous PA on most, preferably all days, of the week [19] was added to the main project theme of '10,000 Steps a Day, Every Step Counts'. A final adaptation in the present study in Flanders was the more general guideline of promoting PA in the entire population and all domains of active living (PA for transport, at work, for household and leisure time), while '10,000 Steps Ghent' and '10,000 Steps Rockhampton' focused on specific projects for primary care, workplaces, and older people (Table 1). This more general recommendation was believed to be less rigid and would facilitate more widespread dissemination and implementation.

### Dissemination efforts for the present study in Flanders

Dissemination efforts for the present study in Flanders (population: 6,160,600 inhabitants, surface: 13,521 square km, density: 456 inhabitants/square km) were initiated at the end of 2007. Considering the limited funding for dissemination (one researcher part-time allocated to the project), the local engagement of potential adopters and the formation of local steering committees could not be established by researchers themselves as was the case in the local projects of '10,000 Steps Rockhampton' and '10,000 Steps Ghent' (Table 1). Dissemination efforts related to the present study in Flanders focused primarily on the use of media and interpersonal contacts with specific professional organizations (e.g. local health promotion services) based on the principles of Rogers' Diffusion of Innovations [7].

A permanent media strategy consisted of a website (updated from '10,000 Steps Ghent') that provided content for both potential adopters and interested citizens. Main web content for potential adopters in Flanders included updated project implementation manuals for the implementation of '10,000 Steps' in communities and workplaces, downloads of intervention materials (e.g. logo, flyers, posters, billboards), project and community contacts, and web space for announcing project activities and events. Main web content for citizens included information about the '10,000 Steps' philosophy, PA and health, tips on how to increase daily activity, contacts, an online diary for recording personal step counts, and reports on planned or completed activities. Two emails containing the publication on the effectiveness of '10,000 Steps Ghent' [13], the project implementation manual and links to the website's content were sent to municipal sports services, local health promotion services, and health insurance organizations. These organizations were identified by contacting their respective national or provincial agency and by consulting interested opinion leaders within the agency.

For the interpersonal component, these opinion leaders subsequently helped analyze peer networks within the organizations by mapping dates, meeting locations and sponsoring organizations of upcoming supra-local group meetings. These group meetings were formal platforms where representatives of the organizations and their peers from other municipalities/provinces regularly joined to discuss professional matters of common interest. A total number of 26 group meetings provided a social platform for presentations with illustrative project materials that accentuated the positive scientific findings of '10,000 Steps Ghent', the compatibility of the current program with the organizations' mission and local contexts, and its easy access through the website. The website and its online support materials for implementing '10,000 Steps' were demonstrated. Present organizations were advised to take advantage of this project support (project implementation manual, downloads of intervention materials, contacts), to form a local steering committee independently, and to implement '10,000 Steps' according to the guidelines and intervention components described in the project implementation manual.

Finally, displays at regional conferences and articles for professional e-magazines also referring to the website were periodically provided between 2007 and the start of data collection.

### Data collection and sample

Data were collected at individual (i.e. citizens) and organizational (i.e. professional organizations) levels.

**Figure 1:**
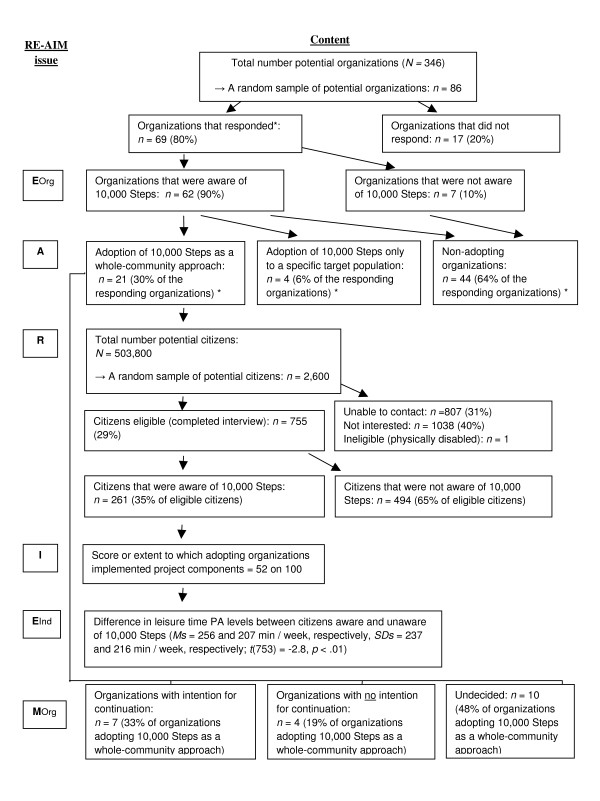
**Flow chart for the different dimensions of the RE-AIM framework applied to the '10,000 Steps' project**. Eorg = effectiveness on the organizational level, R = Reach, A = adoption, I = implementation, Eind = effectiveness on the individual level, M = maintenance.

Individual level measures included telephone interviews (March 2009 to April 2009) where all adult citizens living in the work area of the sample organizations that adopted '10,000 Steps' as a whole-community approach (see measures - *adoption *section) were potential candidates to be included in the study (*N *= 503,800). To establish reach, population telephone registers were consulted and a random sample of 2,600 adults was drawn from the target area of the organizations adopting '10,000 Steps' as a whole-community approach (Figure 1). At least three attempts were made before recording individuals as "unable to contact". This resulted in a total of 755 respondents (29% of the initial sample, response rate 42%) that completed the interview. Citizens were asked to complete the telephone-administered long version of the International Physical Activity Questionnaire (IPAQ) and a questionnaire related to the awareness of '10,000 Steps'.

Organizational level measures were collected using an online survey which was sent to the heads of department of professional organizations (January 2009 to March 2009). As shown in Figure 1, the total number of potential organizations to be included in the study was 346. Of a random sample of 86 contacted organizations, 69 organizations responded (80% response rate).

The study protocols were approved by the Ethical Committee of the Ghent University.

### Measures

Questionnaires (see Additional file 1) assessed individual project awareness and included the following questions: Have you heard of '10,000 Steps'? (yes/no); Where did you hear about '10,000 Steps'? (radio, TV, print media, Internet, street signs or other objects in the street scene, workplace, health service, (para)medic, society, family, friends, other); Are you aware of any of the following activities related to '10,000 Steps'?: sale of pedometers, loan of pedometer, walk circuits between public places or in local town parks? (yes/no). The IPAQ assessed PA in a usual week, including PA at work, transport related PA, domestic and gardening activities, and PA during leisure time. The IPAQ has been proven to be a reliable and valid instrument for assessing PA at the population level in Europe and in Flanders, Belgium [20,21]. Total time for PA in the four domains and total time for walking, moderate, and vigorous PA, all expressed in minutes/week, were computed http://www.ipaq.ki.se.

The online organizational survey (see Additional file 2) was structured in four main parts assessing: 1) an organization's general characteristics and awareness of '10,000 Steps', 2) adoption of '10,000 Steps' and reasons for adopting or declining the project, 3) program implementation, exploring the delivery of the different project components, the frequency of implementation if relevant, resources, targeted domains of active living and project duration, and 4) long-term maintenance of the project.

#### RE-AIM Evaluation

Measures of the RE-AIM dimensions were used to calculate summary metrics and are illustrated in Figure 1.

##### Reach

Reach was defined as the proportion of eligible citizens that reported being aware of '10,000 Steps' (Have you heard of '10,000 Steps'?) [8,17]. Representativeness was estimated by comparing differences in age, gender, education and employment status between those reporting awareness of '10,000 Steps' versus those not aware.

##### Effectiveness

Individual effectiveness compared PA levels of citizens who were aware of '10,000 Steps' (reached individuals) with those who were not aware of it (individuals not reached) and within the four domains of active living (PA for transport, at work, for household and leisure time) [22]. Because an important focus of this dissemination study was to make professional organizations aware of '10,000 Steps' while using limited resources, effectiveness was also measured on the organizational level by comparing the proportion of the studied sample of organizations that was aware of '10,000 Steps' (Is your organization familiar with '10,000 Steps'?) based on prior applications of the RE-AIM framework in studies with smaller settings [23].

##### Adoption

Adoption was evaluated as the proportion and representativeness of organizations that had delivered a whole-community project based on '10,000 Steps' (Did your organization adopt '10,000 Steps' up till this day?). Representativeness was assessed as differences in number of staff members, type of organization and working context *between *the organizations adopting and not adopting '10,000 Steps'[24]. Representativeness of delivery agents *within *adopting organizations was explored by comparing project and non-project staff members on age, years of expertise and gender [9].

##### Implementation

Implementation was measured as the extent to which several intervention components for PA promotion were applied by organizations that adopted '10,000 Steps' as a whole-community approach. These socio-ecological components reflect the key intervention strategies applied in '10,000 Steps Ghent' [13] and also allow for some degree of local adaptation. These components are: 1) the sale or loan of pedometers in public places (Did your organization sell or loan pedometers during the implementation of '10,000 Steps'), 2) use of the '10,000 Steps' website (Did your organization use the supportive website for '10,000 Steps', http://www.10000stappen.be?), 3) repeated dissemination of information using variants of flyers (Did your organization disseminate flyers of '10,000 Steps'?) and 4) posters in public places (Did your organization disseminate posters of '10,000 Steps'?), 5) wide-ranging personal contact with citizens (Did your organization contact citizens in a personalized manner (e.g. personalized letter, mail, or phone)?), 6) the organization of community events (Did your organization stage any community events to promote '10,000 Steps'?), 7) repeated use of the media (Did your organization conduct a media campaign to promote '10,000 Steps'?), 8) the repeated or permanent use of street signs or other strategically placed objects in the street scene (e.g. bill boards) to encourage PA (Did your organization put street signs, billboards, or other promotional materials of '10,000 Steps' in the street scene?) and 9) the initiation of partnerships with local authorities and other associations (Did your organization have any partnerships with municipal services, associations or societies to implement '10,000 Steps'?). As advised by Glasgow and colleagues, the median implementation score (score on 100) across all nine project components was taken as the global implementation score [25]. Organizations acquired a maximum implementation score if a component was recurrently promoted in public places, received half of the maximum score if they presented a component only once, and zero if no component was implemented. Scoring was applied to the following components: pedometer loan or sale, use of the website, dissemination of flyers or variants, posters, community events, use of the media and street signs or similar street scene objects. For the implementation of personal contact, a maximum component score was granted if an attempt was made to contact more than half of the adult citizens through personal correspondence or telephone, half of the score if fewer were contacted, and zero if no efforts were undertaken. For the partnership component a maximum score was obtained when a multidisciplinary partnership was initiated with at least one municipal public service or two other professional organizations, half of the score was obtained if the partnership was less extensive, and zero if no partnership was initiated. Additional measures included the duration and investment of the delivered projects as well as the number of domains of active living that was targeted.

##### Maintenance

The proportion of organizations' intending to continue the delivery of '10,000 Steps' (Does your organization have the intention to continue '10,000 Steps' in the future?) evaluated the potential sustainability of the intervention [26]. Due to the limited time span between the structured dissemination efforts for '10,000 Steps' and the present study's data analysis, it was too early to report data on the potential long-term maintenance at both the individual and organizational level.

### Data analysis

The proportion of citizens aware of '10,000 Steps' was calculated and independent sample *t *tests and chi-square tests were used to compare those aware versus not aware of '10,000 Steps' on demographic characteristics (reach) and for differences in PA levels (effectiveness-individual level). Effect sizes for PA were computed by subtracting mean PA levels of citizens in both groups (citizens aware and not aware), and dividing this score by the pooled standard deviation of PA levels. Effect sizes were interpreted as negligible (< 0.15), small (0.15 - 0.40), medium (0.40 - 0.75) or large (> 0.75) [27].

The proportion of organizations that was aware of '10,000 Steps' (effectiveness-organizational level) and that had delivered '10,000 Steps' (adoption) was calculated. Differences between organizations adopting and not adopting '10,000 Steps', as well as differences between project staff and non-project staff were analyzed with independent sample *t *tests and chi-square tests. Descriptive statistics provided insights in reasons for (not) adopting '10,000 steps' (adoption).

The global implementation score and the implementation scores of the separate project components were calculated and converted to a *z*-score. Descriptive statistics were used to analyze reasons for not implementing components, targeted domains of active living, project duration, and investment (implementation).

The proportion of organizations that had the intention to continue '10,000 Steps' was calculated (maintenance). All analyses were conducted using SPSS 15.0 (SPSS, Inc. Chicago, IL).

## Results

Those responding to the individual survey had a similar proportion of employed people [60.0% vs. 66.3%, respectively; χ^2^(1) = 0.87, *p *= 0.35], a higher mean age [*Ms *= 50.6 years vs. 48.0 years, respectively, *t*(754) = 4.69, *p *< 0.01], a lower proportion of men [40.9% vs. 49.0%, respectively; χ^2^(1) = 19.78, *p *< 0.01], and a higher proportion of persons with a higher education degree [39.7% vs. 25.0%, respectively; χ^2^(1) = 85.18, *p *< 0.01] compared to the general population of Flanders. Responding organizations included 25 municipal sports services (of 42 contacted and of 292 total), 18 local health promotion services (of 18 contacted and of 26 total), and 26 health insurance organizations (of 26 contacted and of 28 total).

Figure 1 presents an overview of the main results for all the RE-AIM dimensions and the next section presents results for each separate dimension.

### Reach

Of those citizens living in the target area of organizations that adopted '10,000 Steps' as a whole-community approach, 35% was aware of '10,000 Steps' (*n *= 261) with no differences found on demographic variables compared to those not aware of '10,000 Steps' (*n *= 494); they had similar mean age [*Ms *= 50.5 years vs. 50.8 years, respectively, *SDs *= 14.5 vs. 16.0, respectively; *t*(576) = 0.29, *p *= 0.66], a similar proportion of men [36.4% vs. 43.3%, respectively; χ^2^(1) = 3.38, *p *= 0.07], a similar proportion of persons with a higher education degree [39.8% vs. 39.6%, respectively; χ^2^(1) = 0.00, *p *= 0.963] and a similar proportion of employed people [77.1% vs. 72.4%, respectively; χ^2^(1) = 1.55, *p *= 0.213].

When considering the most cited information sources about '10,000 Steps', print media was reported by 33% of the reached respondents, followed by TV (21%), health insurance organizations (18%) and friends or family (13%). Furthermore, more than 22% of reached respondents were aware of the sale or promotional distribution of pedometers, 9% was aware of walking circuits, and 3% was aware of a loan system of pedometers.

### Effectiveness

Those individuals aware of '10,000 Steps' reported significantly higher leisure time PA levels than those not aware (see Table 2). No other significant differences were found in the other three PA domains or in the different categories of PA intensity (walking, moderate and vigorous PA).

**Table 2:** Mean physical activity (PA) levels for respondents who were aware and unaware of 10.000 Steps

	*Group aware of 10,000 Steps n = 261*	*Group unaware of 10,000 Steps n = 494*	*df*	*t*	*d*
**Transport-related PA (min/week)**	128 ± 161	116 ± 165	753	-0.9	0.07
**Leisure time PA (min/week)**	256 ± 237	207 ± 216	753	-2.8*	0.22
**Household PA(min/week)**	420 ± 384	412 ± 439	753	-0.3	0.02
**Work-related PA (min/week)**	282 ± 454	310 ± 494	753	0.4	0.07
**Walking (min/week)**	261 ± 289	263 ± 287	753	0.1	0.01
**Moderate PA (min/week)**	667 ± 409	631 ± 400	753	-1.2	0.09
**Vigorous PA (min/week)**	108 ± 203	116 ± 215	753	0.5	0.04

*p ≤ .005

Of the targeted professional organizations 90% (*n *= 62) were aware of '10,000 Steps'.

### Adoption

Of all organizations, 36% reported adoption of '10,000 Steps' (*n *= 25) with 21 (30%) adopting the whole-community approach and four organizations (6%) adapting the approach for specific groups such as personnel of local governments (*n *= 2), the elderly (*n *= 1), and schools (*n *= 1). Comparisons between adopting and non-adopting organizations (*n *= 44) revealed no significant differences in the mean number of staff members [*Ms = *4.1 vs. 5.6, respectively, *SDs *= 2.9 vs. 11.1, respectively; *t*(63) = 0.60, *p *= 0.55], type of organization [76.0% vs. 56.8% with a health policy focus, respectively; χ^2^(1) = 2.53, *p *= 0.11] and working context [16.0% only urban, 16.0% only rural and 68.0% both urban and rural vs. 13.6% only urban, 27.3% only rural and 59.1% both urban and rural, respectively; χ^2^(2) = 1.14, *p *= 0.57].

Delivery agents *within *the adopting organizations showed no significant differences in mean age compared to non-project staff members [*Ms *= 39.7 years vs. 36.4 years, respectively, *SDs *= 8.5 vs. 9.9, respectively; *t*(39) = 1.16, *p *= 0.55]. However, those delivering the program reported more years of experience in PA promotion than non participating staff members [*Ms *= 7.6 vs. 2.9, respectively, *SDs *= 4.6 vs. 5.9, respectively; *t*(36) = 2.79, *p *< 0.01] and had a bigger proportion of women [71.4% vs. 56.8% female, respectively; χ^2^(1) = 4.06, *p *< 0.05].

Non-adopting organizations listed reasons for having doubts or not having the intention to adopt '10,000 Steps'. These were: 'not having thought about it yet in a concrete manner' (70%), 'insufficiently planned resources' (30%), 'no priority or not suitable' (20%) and 'need for knowledge support and good-practices' (15%). Adopting organizations and those that had the intention to do so in the future gave the following main reasons for adoption: '10,000 Steps' is a ready-for-use product' (40%), 'assignment given by the organization's superior' (35%), 'the scientific evidence of the pilot project' (26%) and 'experience of peers in other organizations' (19%).

### Implementation

The global implementation score was a median 52 of 100. Four of nine separate components gained less than half of the maximum implementation score (see Table 3 for corresponding *z*-scores). Most reported reasons for not having implemented these components are also indicated in Table 3.

**Table 3:** Implementation scores for each project component and reasons for not having implemented them

*Project component*	*Implementation score on 100 (mean ± SD)*	*z-score*	Three most mentioned reasons for not having implemented the component (% of organizations)*
Sale or loan of pedometers	90.5 ± 30.7	1.33	
Organization of community events	87.5 ± 34.2	1.22	
Dissemination of information using variants of flyers	76.2 ± 34.0	0.82	
Use of the media to promote '10,000 Steps'	64.3 ± 42.3	0.39	
Use of the '10,000 Steps' website	52.4 ± 51.2	-0.04	
Initiation of partnerships with local authorities and other associations	37.5 ± 45.5	-0.57	No time (36%) No added value for the project (27%) Not relevant to our core business (27%)
Use of posters in public places	35.7 ± 45.1	-0.63	Need for more information with regard to content or support (55%) Not relevant to our core business (45%) Still considering implementation (9%)
Use of street signs or other strategically placed objects in the street scene	19.0 ± 37.0	-1.23	Need for more information with regard to content or support (55%) Not relevant to our core business (45%) Too expensive (9%)
Wide-ranging personal contact with citizens	17.2 ± 28.5	-1.30	No time (33%) Not relevant to our core business (33%) Too expensive (17%)

*Global implementation score (median)*	*52.4*		

* This is only indicated for components with mean implementation scores < 50 on 100

Almost half of the organizations reported a project duration of more than one year (48%; *n *= 10). Fourteen percent reported a project duration of 7 to 12 months (*n *= 3), 24% a duration of 2 to 6 months (*n *= 5) and 14% a duration of one month or less (*n *= 3).

When considering the targeted domains of active living or PA contexts, leisure time was marked most frequently by 95% of the organizations (*n *= 20), followed by active transport (91%; *n *= 19), household activities (81%; *n *= 17), and work-related activities (72%; *n *= 15).

Organizations devoted an average of 47 (± 72) working days on '10,000 Steps'. They estimated a financial investment of 0.026 (*SD *= 0.018) euros (or 0.032 (SD = 0.022) dollars) per citizen for project implementation.

### Maintenance

Of all the organizations that adopted '10,000 Steps' as a whole-community approach, 33% (*n *= 7) had the intention to continue the project in the future. An additional 48% was still undecided (*n *= 10), while 19% (*n *= 4) had no intention to continue the project.

## Discussion

The current study applied the RE-AIM framework to the evaluation of the wide-scale dissemination of '10,000 Steps' beyond initial applications ('10,000 Steps Ghent'). The dissemination effort was highly effective in making professional organizations aware of '10,000 Steps' (90%), with lower scores for adoption (36%), implementation (52%), and the intention of project continuation (33% and an additional 48% of undecided organizations).

The combination of media strategies and interpersonal contact (i.e. peer networks) through supra-local meetings with organizational representatives and opinion leaders seems promising for creating project awareness of '10,000 Steps' among a substantial proportion of organizations. Diffusion theory supports the current dissemination approach, that relied on media to have an impact in creating knowledge, persuasion and decision, and on interpersonal contact and social networks to be influential in the final stages of trial and adoption of an innovation [6,7]. However, adoption was problematic since only about one third of the studied organizations adopted '10,000 Steps'. According to diffusion theory this may be due to the need for additional time to allow the adoption to occur through the different stages of the innovation-decision process [28]. This reasoning is supported by the high proportion of non-adopting organizations in this study (70%) that reported 'not having thought about adoption yet in a concrete manner'. Similarly, the state-wide dissemination of the 'Walk Kansas' project showed a time dependent factor, with moderate adoption in its first year and changing adoption rates in the following years [29]. High external validity was found, with adoption rates in the present study found to be independent of organizational characteristics such as staff size, type of organization, working context, and age of staff.

The modest global implementation score was caused primarily by disappointing implementation scores on separate components including partnerships with local authorities and other associations, high visibility components such as posters in public places and variants of street signs, and wide-ranging personal contact with citizens. Community partnerships have proven their importance for effective health and PA promotion before [30,31] and have been recommended as a reinforcing component in other large-scale national PA programs [32,33]. Within the most often mentioned reasons for not initiating partnerships, organizations listed 'no time' and 'no added value'. These findings show similarities with previous reviews of partnerships and experiences in recent community studies reporting the time-consuming and complex formation of multidisciplinary partnerships for PA promotion, as well as the difficulty of engaging stakeholders to invest resources into a PA project that is not considered as their "core business" [32,34,35]. The relative importance of high visibility components such as street signs or similar adaptations has been demonstrated in '10,000 Steps Ghent'. Mediation analysis showed that the positive behavioural outcomes of '10,000 Steps Ghent' were significantly mediated through citizens' awareness of street signs and workplace projects [36]. The most frequent reported barriers for street signs or variants in this study ('need for more information with regard to content or support' and 'not relevant to our core business') indicate that more efforts are required to inform professional organizations about this component, to support its application, and to stimulate ecologic (multi-strategy and intersectoral) insights in organizations' PA policy. Wide scale personal contact with the target population proved to be the least feasible project component, 'having no time' and 'not relevant to our core business' being the most reported barriers. Only one organization achieved a maximum score on this component. However, additional research using more structural support could address the question of whether implementation of this component could be enhanced.

Additional research will also allow forming more conclusive insights regarding maintenance (and local adaptation) of '10,000 Steps'. More than one of three adopting organizations had the intention to continue the project, but an even greater number (more than 4 of 10) was still undecided. The latter finding also represents an opportunity for project advocates to increase maintenance of '10,000 Steps', e.g. by providing supportive services or the encouragement of multi-disciplinary work-groups which could foster project maintenance [37].

On the individual level, we found a moderate reach or awareness of '10,000 Steps', and those aware of the project reported higher PA levels during leisure time activities compared to those not aware. Citizens' awareness was representative on all studied demographic characteristics. Nevertheless, the proportion of citizens that was aware of '10,000 Steps' in this study (35%) was considerably lower than in the studies of '10,000 Steps Ghent' (63%) and '10,000 Steps Rockhampton (90%) [11,13]. The most cited information sources about '10,000 Steps' in the present study were similar to the ones in '10,000 Steps Ghent', confirming the general potential of media (print media and TV) and health insurance organizations as informational conduits. However, in contrast with the pilot study, street signs and the work place were not as frequently reported in the present study. In the present study these two information sources represented the least implemented project component (street signs) and PA context (work place). Consistent with socio-ecological principles of health promotion, these findings could imply that environmental strategies such as street signs and organizational strategies such as work place interventions are recommended to increase project awareness [18].

Implementation outcomes may also be associated with particular findings on PA levels. The leisure time context of PA was targeted more intensively by almost all the organizations in the present study and may be related to the significant differences in PA levels during leisure time activities (small but considerable effect sizes for a whole-community project) of citizens.

It appears that '10,000 Steps' has good potential for wide-scale dissemination in particular with the addition of more in-depth information to organizations and a more effective support structure to encourage project adoption and full implementation. Implementation support was limited to a centralized website, which was probably insufficient to fully support translation of a whole-community project like '10,000 Steps' into practice. According to Green and colleagues a centralized approach is unlikely to fill the gap between the centralized supply side and the more local demand sides of science [6]. A more participatory approach using a linkage system that connects the developers of a project to the potential users may be a better instrument and has been argued to stimulate the process of diffusion, adoption and implementation [38-40]. Since this research ended, funding of the Flemish Government is supporting an intermediate structure of linkage agents so future data on this structure is forthcoming.

Limitations of the current results are noted. Generalizability of results may be limited because no more than half of the contacted citizens agreed to participate and no information was available about the characteristics of the non-participants. This could have led to a positive bias of the results on citizens' project awareness and PA levels. Sole use of self-reported data was another limitation. Due to social desirability this may also have led to a positive bias of the results on project implementation (organizational level) and PA levels (individual level). On the organizational level, self-reported data on project implementation could become more valid by adding objective measures [9], such as on-site observations by a team of researchers (which require more resources than available in this study). On the individual level, complementary objective measures of PA levels could include the use of pedometers or accelerometers. Nevertheless, the questionnaire to collect data on PA levels in this study (telephone-administered IPAQ) has proven acceptable validity against accelerometers in several countries [20]. Finally, the study's outcomes on maintenance are provisional because these are based on adopters' intentions and not on actual program continuation. Follow-up research is already scheduled to study program continuation and implementation one year after the present study.

Strengths of this research include the multi-site study of large scale project dissemination without using convenience sampling for selecting the sites. Other multi-site community-based studies often use this method on the basis of site interest, resources or proximity, which limits generalizability of results [9]. Second, the present study also adds data to the still modest knowledge base of information on whole-community PA programs' potential to be adopted and implemented within existing delivery systems. In Australia, a preliminary dissemination of '10,000 Steps' was studied using the internet as a means of disseminating project resources [41]. However, in contrast to our study, the Australian study did not report on the proportion and representativeness of adopting organizations or on the quality of implementation, which makes it more difficult to make comparisons. Third, the present study reported additional qualitative data related to adoption and implementation, which provided a better understanding of scores on these dimensions. Fourth, an effectiveness dimension on the organizational level was added. This provided an answer to the question if efforts to make organizations aware of '10,000 Steps' were effective. It also provided complementary information on adoption rates and to which extent these rates were mediated by the applied dissemination strategy or by the characteristics of the project itself.

## Conclusions

In conclusion, '10,000 Steps' shows potential for wide-scale dissemination but a more extensive support structure than the one applied in the present study seems recommended to encourage adoption levels and high quality implementation. Follow-up research that integrates such a structure will provide more conclusive insights, also regarding the potential of '10,000 Steps' for program continuation (sustainability).

## Competing interests

The authors declare that they have no competing interests.

## Authors' contributions

RVA designed the study, collected and analyzed the data, and drafted the manuscript. IDB participated in the design of the study and helped to draft the manuscript. KDC and LK also helped to draft the manuscript and interpret data. GC conceived of the study, participated in the design, and helped to draft the manuscript. All authors read and approved the final manuscript.

## Pre-publication history

The pre-publication history for this paper can be accessed here:

http://www.biomedcentral.com/1471-2458/11/3/prepub

## Supplementary Material

Additional file 1 **Questionnaire for citizens**. Questionnaire_citizens_10000Steps.pdf Questionnaire to assess individual project awareness and PA levels (including IPAQ). Click here for file

Additional file 2 **Organizational survey**. Organizational_survey_10000Steps.pdf Survey to assess organizational project awareness, adoption, implementation and long-term maintenance of '10,000 Steps'. Click here for file
