# Supplementary material for: The impact of disseminating the whole-community project '10,000 Steps': a RE-AIM analysis
Source: BMC Public Health. 2011 Jan 4;11:3. doi: 10.1186/1471-2458-11-3 (PMC3022698; doi:10.1186/1471-2458-11-3)
Supplement: Additional file 1 — Questionnaire for citizens. Questionnaire_citizens_10000Steps.pdf Questionnaire to assess individual project awareness and PA levels (including IPAQ). [file 1471-2458-11-3-S1.PDF]

39151

ID nr | | | |

Vragenlijst: interview 10.000 stappen

LEES: Goeiedag, Mr./Mevr., u spreekt met (*naam interviewer*), medewerker van Universiteit Gent bij de vakgroep Bewegingswetenschappen.

Universiteit Gent voert een studie uit naar uw gemeente om na te gaan of zij projecten rond lichaamsbeweging voldoende bij de inwoners bekend maakt. We bellen telkens 40 inwoners op per gemeente, en u bent voor (*naam gemeente*) één hiervan.

Graag zouden we dan ook enkele vragen aan u voorleggen. Dit zal +-10 minuten in beslag nemen. Kan u hier even tijd voor vrij maken?

(**Interviewer verduidelijking:** antwoorden zullen strikt vertrouwelijk behandeld worden)

Perceptie van "10.000 stappen"

1. Hoorde of zag u het afgelopen jaar boodschappen in verband met beweging, fysieke activiteit? (kleur **1** bolletje)

- O Neen *(ga naar vraag 2)*
- O Ja

Indien ja, kan u daar kort iets over vertellen?

-Wat?

-Waar?

-Wanneer?

39151

2. Hoeveel beweging is nodig om gezondheidsvoordelen te bekomen volgens u? (kleur **1** bolletje - vul aan indien nodig)

- O Ik heb geen idee
- O Ik denk:

3. Hoorde of zag u het afgelopen jaar informatie over "10.000 stappen"? (kleur **1** bolletje)

- O Neen *(ga naar vraag 7)*
- O Ja

4. Waar hoorde of zag u informatie over "10.000 stappen"? (kleur **1** of **meer** bolletjes)

- O Radio
- O Televisie
- O Geschreven pers
- O Internet
- O Straatbeeld
- O Werk/bedrijf
- O Andere - verduidelijk:
- O Arts
- O Kinesist / kine
- O Diëtist
- O Sportdienst
- O Clubhuis senioren
- O Ziekenhuis
- O Sportclub / wandelclub
- O Socioculturele vereniging
- O Familie
- O Vrienden
- O School van uw kinderen
- O Dienstencentra of OCMW
- O Mutualiteit / Ziekenfonds

5. Van welke acties van "10.000 stappen" bent u op de hoogte? (kleur **1** of **meer** bolletjes)

Verkoop stappentellers

- O ...in apotheek
- O ...in sportdienst van de gemeente
- O ...in ziekenfonds / mutualiteit
- O ...Ander - verduidelijk:

Mogelijkheid tot uitlenen van stappentellers

- O ...in sportdienst van de gemeente
- O ...in bibliotheek
- O ...Ander - verduidelijk:

O Receatieve "10.000 stappen"-wandelparcours (bv. in recreatiedomeinen, parken...)

O Wandelmogelijkheden tssn publieke plaatsen in gemeente (tssn gemeentehuis en bib/parkings...)

O Ander - verduidelijk:

6. In welke mate bent u het er mee eens dat de info en/of acties rond "10.000 stappen" u persoonlijk hebben aangezet om uw beweging te vermeerderen? (kleur **1** bolletje)

Helemaal niet akkoord	Niet akkoord	Soms wel/niet akkoord	Eerder wel akkoord	Helemaal akkoord
O	O	O	O	O

Pedometergebruik

7. Heeft u het afgelopen jaar een pedometer gebruikt om uw stappen bij te houden? (kleur **1** bolletje)

O Neen *(ga naar vraag 14)*

O Ja *(ga naar vraag 8)*

39151

8. Waar haalde u uw pedometer? (kleur **1** of meerdere bolletjes)

- O Gekocht bij gemeentelijke diensten (bv. gemeentelijke sportdienst...)
- O Gekocht bij de apotheker
- O Gekocht bij ziekenfonds
- O Geleend bij gemeentelijke diensten (bv. sportdienst...)
- O Geleend bij familie
- O Geleend bij vrienden
- O Ander - verduidelijk:

9. Hoe vaak draagt u een pedometer tegenwoordig? (kleur **1** bolletje)

- O Elke dag *(ga naar vraag 12)*
- O Enkele keren per week *(ga naar vraag 12)*
- O Enkele keren per maand *(ga naar vraag 12)*
- O Af en toe *(ga naar vraag 12)*
- O Nooit *(ga naar vraag 10)*

10. Als u tegenwoordig geen pedometer draagt, hoelang droeg u hem in het verleden? (kleur **1** bolletje)

- O Enkele dagen
- O Een week
- O Minstens 6 maanden
- O Minstens een jaar

11. Hoe dikwijls was dat toen? (kleur **1** bolletje)

- O Elke dag
- O Enkele keren per week
- O Enkele keren per maand
- O Af en toe
- O Nooit

12. Op een gemiddelde dag, hoeveel stappen per dag zet/zette u gemiddeld als u een stappenteller draagt/droeg? (kleur **1** bolletje)

- O minder dan 5000 stappen/dag
- O tussen 5000 en 7499 stappen/dag
- O tussen 7500 en 9999 stappen/dag
- O tussen 10000 en 12499 stappen/dag
- O meer dan 12500 stappen/dag

39151

13. In welke mate bent u het er mee eens dat een pedometer u helpt/hielp om uw beweging te vermeerderen? (kleur **1** bolletje)

Helemaal niet akkoord	Niet akkoord	Soms wel/niet akkoord	Eerder wel akkoord	Helemaal akkoord
O	O	O	O	O

Website: perceptie en gebruik

14. Kent u de website van "10.000 stappen", namelijk www.10000stappen.be? (kleur **1** bolletje)

O Neen *(ga naar vraag 16)*

O Ja, deze heb ik 1 keer gebruikt/bezocht

O Ja, deze heb ik meermaals gebruikt/bezocht

O Ja, deze heb ik meermaals gebruikt/bezocht en ook het elektronisch stappenboek ingevuld

15. In welke mate bent u het er mee eens dat de info op deze website nuttig is? (kleur **1** bolletje)

Helemaal niet akkoord	Niet akkoord	Soms wel/niet akkoord	Eerder wel akkoord	Helemaal akkoord
O	O	O	O	O

Demografische info

16a. Wat is uw voornaam?

16b. Wat is uw (achter)naam?

17. Wat is uw telefoonnummer?

18. Wat is uw geslacht ? (kleur **1** bolletje)

O man

O vrouw

19. Hoe oud bent u nu ? [ ][ ] jaar

39151

20. Wat is het hoogste diploma of getuigschrift dat u behaald heeft (of momenteel bezig bent te behalen) ? (kleur **1** bolletje)

- O lager onderwijs
- O beroepssecundair onderwijs (BSO)
- O technisch secundair onderwijs (TSO)
- O algemeen secundair onderwijs (humaniora, kunstonderwijs, ...)
- O hoger onderwijs buiten de universiteit
- O universitair onderwijs

21. Wat is uw huidige werksituatie ? (kleur **1** bolletje)

- O onderwijs
- O bediende
- O kaderpersoneel
- O zelfstandige (geen vrij beroep)
- O vrij beroep
- O arbeider
- O student
- O geen beroep

22. Hoe zou u uw algemene gezondheid omschrijven? ? (kleur **1** bolletje)

- O Uitstekend
- O Zeer goed
- O Goed
- O Matig
- O Zwak

23. Bij welk ziekenfonds / mutualiteit bent u aangesloten? (kleur **1** of meerdere bolletjes)

(**Interviewer verduidelijking:** We stellen deze vraag om te weten of info van ziekenfondsen omtrent lichaamsbeweging tot bij de leden raakt.)

- O Bond Moyson / De Voorzorg / Socialistische Mutualiteiten
- O CM (Christelijke Mutualiteiten)
- O Liberale Mutualiteiten
- O Securex
- O Partena
- O Euromut
- O Onafhankelijk Ziekenfonds (OZ)
- O Vlaams en Neutraal Ziekenfonds

Uw fysieke activiteit

LEES: Ik ga u nu enkele vragen stellen over uw fysieke activiteit of lichaamsbeweging in een doorsnee week.

Deel 1: Fysieke activiteiten tijdens uw werk

LEES: De eerste vragen gaan over uw werk. Dit kan betaald werk zijn, maar ook onbetaald werk zoals, vrijwilligerswerk of studiewerk. Huishoudelijk werk, tuinieren, en klusjes horen hier *niet* bij.

24. Hebt u momenteel een baan of doet u onbetaald werk buitenshuis ?

- O ja
- O nee (Ga naar Deel 2: Vervoer)

(**Interviewer verduidelijking:** 'werk' omvat ook nascholingen en werk voor cursussen volwassenenonderwijs. Dit omvat ook vrijwilligerswerk en de tijd op zoek naar werk. Het omvat niet huishoudelijk werk, tuinieren, of gezinszorg, dit wordt gevraagd in een later deel)

LEES: De volgende vragen gaan over alle fysieke activiteiten die u tijdens een gewone week verricht als deel van uw betaald of onbetaald werk. De verplaatsing van en naar het werk hoort hier *niet* bij.

LEES: Denk eerst aan alle zware fysieke activiteiten op uw werk. Deze vragen een lichamelijke inspanning waarbij u veel sneller en dieper ademt dan normaal. Denk alleen aan activiteiten die u gedurende minstens 10 minuten aan één stuk doet

25. Op hoeveel dagen in een gewone werkweek doet u zware fysieke activiteiten zoals zwaar tilwerk, spitten, bouwwerken of trappen oplopen *als deel van uw werk* ?

☐ dagen per week

O Geen (Ga naar vraag 27)

(**Interviewer verduidelijking:** denk *alleen* aan die fysieke activiteiten die u gedurende minstens 10 minuten aan één stuk verricht)

26. Hoeveel tijd in totaal besteedt u op zo'n dag gewoonlijk aan zware fysieke activiteiten *als deel van uw werk* ?

☐ uur ☐☐ minuten/dag

(**Interviewer verduidelijking:** denk *alleen* aan die fysieke activiteiten die u gedurende minstens 10 minuten aan één stuk verricht)

39151

LEES: Denk nu aan alle matige fysieke activiteiten op uw werk. Deze vragen een lichamelijke inspanning waarbij u iets sneller en dieper ademt dan normaal. Ook hier, denk alleen aan activiteiten die u gedurende minstens 10 minuten aan één stuk doet.

27. Op hoeveel dagen in een gewone werkweek doet u matige fysieke activiteiten zoals het dragen van lichte lasten *als deel van uw werk*?

☐ dagen per week

○ Geen (Ga naar vraag 29)

(**Interviewer verduidelijking:** denk *alleen* aan die fysieke activiteiten die u gedurende minstens 10 minuten aan één stuk verricht)

(**Interviewer verduidelijking:** werk omvat betaald en onbetaald werk alsook studiewerk. Breng alle jobs in rekening)

28. Hoeveel tijd in totaal besteedt u op zo'n dag gewoonlijk aan matige fysieke activiteiten *als deel van uw werk*?

☐ uur ☐☐ minuten/dag

(**Interviewer verduidelijking:** denk *alleen* aan die fysieke activiteiten die u gedurende minstens 10 minuten aan één stuk verricht)

LEES: Denk nu aan de tijd besteed aan wandelen op uw werk. De verplaatsing te voet van en naar het werk hoort hier niet bij.

29. Op hoeveel dagen in een gewone werkweek wandelt u gedurende minstens 10 minuten aan één stuk *als deel van uw werk ?*

☐ dagen per week

○ Geen (Ga naar Deel 2: Vervoer)

30. Hoeveel tijd in totaal wandelt u op zo'n dag *als deel van uw werk*?

☐ uur ☐☐ minuten/dag

31. Indien u wandelt *als deel van uw werk*, in welk tempo is dat dan meestal ? Wandelt u in :

- ○ een hoog tempo?
- ○ een middelmatig tempo?
- ○ een laag tempo?

Deel 2: Fysieke activiteiten die verband houden met vervoer

LEES: Denk nu aan de manier waarop u zich verplaatst naar het werk, om boodschappen te doen, naar de film te gaan enzovoort.

32. Op hoeveel dagen in een gewone week verplaatst u zich *met een motorvoertuig zoals de trein, de bus, de wagen of de tram* ?

☐ dagen per week

○ Geen (Ga naar vraag 34)

33. Hoeveel tijd in totaal besteedt u op zo'n dag gewoonlijk aan verplaatsingen *met de wagen, de bus, de trein of een ander motorvoertuig* ?

☐ uur ☐☐ minuten/dag

LEES: Denk nu alleen aan het *fietsen en het wandelen* dat u doet om naar het werk te gaan, te winkelen of gewoon om ergens heen te gaan.

34. Op hoeveel dagen in een gewone week fietst u gedurende minstens 10 minuten aan één stuk *om ergens heen te gaan* ?

☐ dagen per week

○ Geen (Ga naar vraag 37)

35. Hoeveel tijd in totaal fietst u op zo'n dag *om ergens heen te gaan* ?

☐ uur ☐☐ minuten/dag

(**Interviewer verduidelijking**: denk *alleen* aan het fietsen dat u gedurende minstens 10 minuten aan één stuk doet)

39151

36. Als u *zich verplaatst per fiets*, in welk tempo fietst u dan meestal ?
Fietst u in :

- O een hoog tempo
- O een middelmatig tempo
- O een laag tempo

37. Op hoeveel dagen in een gewone week wandelt u gedurende minstens 10 minuten aan één stuk *om ergens heen te gaan* ?

☐ dagen per week

O Geen (Ga naar Deel 3: Huishoudelijk werk, klusjes en gezinstaken)

38. Hoeveel tijd in totaal wandelt u op zo'n dag *om ergens heen te gaan* ?

☐ uur ☐☐ minuten/dag

(**Interviewer verduidelijking:** denk *alleen* aan het wandelen dat u gedurende minstens 10 minuten aan één stuk doet)

39. Als u wandelt *om ergens heen te gaan*, in welk tempo is dat dan meestal ?
Wandelt u in :

- O een hoog tempo
- O een middelmatig tempo
- O een laag tempo

Deel 3: Huishoudelijk werk, klusjes en gezinstaken

LEES: Denk nu aan de fysieke activiteiten die u tijdens een gewone week doet in en rond het huis, bijvoorbeeld huishoudelijk werk, tuinieren, klusjes of voor het gezin zorgen.

LEES INDIEN NOG NIET AAN BOD GEKOMEN BIJ WERK: Denk eerst aan alle zware fysieke activiteiten. Deze vragen een lichamelijke inspanning waarbij u veel sneller en dieper ademt dan normaal.

LEES: Nogmaals, denk alleen aan die fysieke activiteiten die u gedurende minstens 10 minuten aan één stuk verricht.

40. Op hoeveel dagen in een gewone week doet u zware fysieke activiteiten zoals zwaar tilwerk, houthakken, sneeuwruimen of spitten *in de tuin of moestuin*.

☐ dagen per week

○ Geen (Ga naar vraag 3c)

(**Interviewer verduidelijking indien nodig**: Zware fysieke activiteiten vragen een lichamelijke inspanning waarbij u veel sneller en dieper ademt dan normaal.

41. Hoeveel tijd in totaal besteedt u op zo'n dag gewoonlijk aan zware fysieke activiteiten *in de tuin of moestuin* ?

☐ uur ☐☐ minuten/dag

(**Interviewer verduidelijking:** denk *alleen* aan die fysieke activiteiten die u gedurende minstens 10 minuten aan één stuk verricht.)

LEES INDIEN NOG NIET AAN BOD GEKOMEN BIJ WERK: Denk nu aan alle matige fysieke activiteiten. Deze vragen een lichamelijke inspanning waarbij u iets sneller en dieper ademt dan normaal. Ook hier, denk alleen aan activiteiten die u gedurende minstens 10 minuten aan één stuk doet.

42. Op hoeveel dagen in een gewone week doet u matige fysieke activiteiten zoals lichte lasten dragen, ruiten wassen, vegen of harken *in de tuin of moestuin* ?

☐ dagen per week

○ Geen (Ga naar vraag 44)

(**Interviewer verduidelijking:** denk *alleen* aan die fysieke activiteiten die u gedurende minstens 10 minuten aan één stuk verricht.)

(**Interviewer verduidelijking indien nodig**: Interviewer verduidelijking indien nodig: matige fysieke activiteiten vragen een lichamelijke inspanning waarbij u iets sneller en dieper ademt dan normaal)

43. Hoeveel tijd in totaal besteedt u op zo'n dag gewoonlijk aan matige fysieke activiteiten *in de tuin of moestuin* ?

☐ uur ☐☐ minuten/dag

(**Interviewer verduidelijking:** denk *alleen* aan die fysieke activiteiten die u gedurende minstens 10 minuten aan één stuk verricht.)

44. Op hoeveel dagen in een gewone week doet u matige fysieke activiteiten zoals lichte lasten dragen, ruiten wassen, vloeren schrobben of vegen *binnenshuis*?

☐ dagen per week

○ Geen (Ga naar Deel 4)

(**Interviewer verduidelijking:** denk *alleen* aan die fysieke activiteiten die u gedurende minstens 10 minuten aan één stuk verricht.)

(**Interviewer verduidelijking:** Op hoeveel dagen in een gewone week doet u fysieke activiteiten binnenshuis die tenminste matig intensief zijn?)

45. Hoeveel tijd in totaal besteedt u gewoonlijk op zo'n dag aan matige fysieke activiteiten *binnenshuis*?

☐ uur ☐☐ minuten/dag

(**Interviewer verduidelijking:** denk *alleen* aan die fysieke activiteiten die u gedurende minstens 10 minuten aan één stuk verricht.)

Deel 4: Fysieke activiteiten die verband houden met sport, ontspanning en vrije tijd

LEES: Denk nu aan alle fysieke activiteiten die u tijdens een gewone week doet, maar dan uitsluitend als recreatie, sport, training of vrijetijdsbesteding. Gelieve geen activiteiten mee te rekenen die u reeds vermeld hebt.

46. Zonder het wandelen dat u reeds vermeld hebt, op hoeveel dagen in een gewone week wandelt u gedurende minstens 10 minuten aan één stuk *in uw vrije tijd*?

☐ dagen per week

○ Geen (Ga naar vraag 49)

47. Hoeveel tijd wandelt u in totaal op zo'n dag *in uw vrije tijd*?

☐ uur ☐☐ minuten/dag

(**Interviewer verduidelijking:** denk *alleen* aan het wandelen dat u gedurende minstens 10 minuten aan één stuk verricht.)

48. Als u wandelt *in uw vrije tijd*, in welk tempo is dat dan meestal ? Wandelt u in :

○ een hoog tempo
○ een middelmatig tempo
○ een laag tempo

39151

49. Op hoeveel dagen in een gewone week doet u <u>zware</u> fysieke activiteiten zoals bijvoorbeeld aerobics, lopen of snel fietsen of snel zwemmen, *in uw vrije tijd* ?

☐ dagen per week

○ Geen (Ga naar vraag 51)

(**Interviewer verduidelijking indien nodig**: Zware fysieke activiteiten vragen een lichamelijke inspanning waarbij u veel sneller en dieper ademt dan normaal.

(**Interviewer verduidelijking:** denk *alleen* aan die fysieke activiteiten die u gedurende minstens 10 minuten aan één stuk verricht.)

50. Hoeveel tijd in totaal besteedt u op zo'n dag gewoonlijk aan zware fysieke activiteiten *in uw vrije tijd* ?

☐ uur ☐☐ minuten/dag

(**Interviewer verduidelijking:** denk *alleen* aan die fysieke activiteiten die u gedurende minstens 10 minuten aan één stuk verricht.)

51. Op hoeveel dagen in een gewone week doet u matige fysieke activiteiten zoals bijvoorbeeld fietsen aan een middelmatig tempo, zwemmen aan een middelmatig tempo, of tennis dubbelspel *in uw vrije tijd* ?

☐ dagen per week

○ Geen (Ga naar Deel 5: De tijd die u zittend doorbrengt)

(**Interviewer verduidelijking indien nodig**: Matige fysieke activiteiten vragen een matige lichamelijke inspanning waarbij u iets sneller en dieper ademt dan normaal

(**Interviewer verduidelijking:** denk *alleen* aan die fysieke activiteiten die u gedurende minstens 10 minuten aan één stuk verricht.)

52. Hoeveel tijd in totaal besteedt u op zo'n dag gewoonlijk aan matige fysieke activiteiten *in uw vrije tijd* ?

☐ uur ☐☐ minuten/dag

(**Interviewer verduidelijking:** denk *alleen* aan die fysieke activiteiten die u gedurende minstens 10 minuten aan één stuk verricht.)

39151

Deel 5: De tijd die u zittend doorbrengt

LEES: Tenslotte zouden we meer willen weten over de tijd die u elke dag zittend doorbrengt op het werk, thuis, tijdens studiewerk of in uw vrije tijd. Hierbij hoort ook de tijd dat u achter een bureau zit, bezoek krijgt, zit te lezen, of naar televisie zit of ligt te kijken.

De tijd die u zittend doorbrengt in een motorvoertuig, die u reeds vermeld hebt, komt hier niet in aanmerking.

53. Hoeveel tijd in totaal brengt u gewoonlijk *zittend* door op een weekdag ?

☐☐ uur ☐☐ minuten/dag

(**Interviewer verduidelijking:** reken de tijd mee die u liggend (wakker zijnde) of zittend doorbrengt)

54. Hoeveel tijd in totaal brengt u gewoonlijk *zittend* door op een weekenddag ?

☐☐ uur ☐☐ minuten/dag

(**Interviewer verduidelijking:** reken de tijd mee die u liggend (wakker zijnde) of zittend doorbrengt)

Dank voor uw medewerking!

39151
