# Supplementary material for: The impact of disseminating the whole-community project '10,000 Steps': a RE-AIM analysis
Source: BMC Public Health. 2011 Jan 4;11:3. doi: 10.1186/1471-2458-11-3 (PMC3022698; doi:10.1186/1471-2458-11-3)
Supplement: Additional file 2 — Organizational survey. Organizational_survey_10000Steps.pdf Survey to assess organizational project awareness, adoption, implementation and long-term maintenance of '10,000 Steps'. [file 1471-2458-11-3-S2.PDF]

UNIVERSITEIT GENT

Faculteit Geneeskunde en Gezondheidswetenschappen
Vakgroep Bewegings- en Sportwetenschappen
Watersportlaan 2
9000 Gent

Steunpunt beleidsrelevant onderzoek 2007-2011 cultuur, jeugd en sport

We stellen het ten zeerste op prijs dat u bereid bent om te helpen bij de implementatiestudie van “10 000 Stappen”.

Uw antwoorden op de vragen in deze vragenlijst zijn zeer belangrijk voor de Vlaamse overheid en uw organisatie. Zij zullen bijdragen tot inzichten en aanbevelingen om projecten zoals “10 000 Stappen” beter af te stemmen op uw ziekenfonds.

Het afwerken van deze online vragenlijst zal +- 8 tot 30 minuten in beslag nemen.

Vergeet niet:

- wij willen weten wat u denkt
- er zijn geen goede of slechte antwoorden
- uw antwoorden zullen strikt vertrouwelijk behandeld worden

Bedankt voor uw deelname aan de implementatiestudie van “10 000 Stappen”!

Vriendelijke groeten,

Mast. en Lic. Ragnar Van Acker

Prof. Ilse De Bourdeaudhuij

Prof. Greet Cardon

Algemene informatie

1. Bij welke organisatie of dienst werkt u? (*kruis **1** bolletje aan*)

O Gemeentelijke/stedelijke (sport)dienst
O Ziekenfonds
O Logo
O Ander: ...

2. In welk type gebied is uw GVO-dienst(*) of dienst gezondheidspromotie actief? (*kruis **1** bolletje aan*)

O In zowel stedelijk als landelijk gebied
O Enkel in stedelijk gebied
O Enkel in landelijk gebied

** GVO: dienst: dienst Gezondheidsvoorlichting en -opvoeding*

3. Hoeveel vaste werknemers telt uw GVO-dienst of dienst gezondheidspromotie? (*kruis **1** bolletje aan*)

O 1 vaste werknemer
O 2 tot 5 vaste werknemers
O 6 tot 10 vaste werknemers
O 11 tot 15 vaste werknemers
O 16 of meer vaste werknemers

4. Is uw GVO-dienst of dienst gezondheidspromotie op de hoogte van het project ‘10.000 Stappen’?

O Neen **(*voor u eindigt de vragenlijst hier*)**
O Ja ***(→ga naar vraag 5)***

Indien u zonet ‘Neen’ heeft geantwoord op deze vraag 4, <u>eindigt de vragenlijst hier</u>.
Dank voor uw medewerking!

5. Sinds wanneer is uw GVO-dienst of dienst gezondheidspromotie op de hoogte van '10.000 Stappen'? (*kruis **1** bolletje aan*)

- O sinds minder dan 6 maanden
- O sinds 6 maanden tot 1 jaar
- O sinds 1 tot 1,5 jaar
- O sinds meer dan 2 jaar

6. Langs welk kanaal is uw GVO-dienst of dienst gezondheidspromotie geïnformeerd over '10.000 Stappen'? *(kruis **1** of **meerdere** bolletjes aan)*

- O via collega-organisaties uit een andere regio/ gemeente
- O via het Interlogo
- O via de centrale ziekenfondszetel
- O via Universiteit Gent of Steunpunt Cultuur, Jeugd en Sport
- O via de Provincie
- O via het ISB (congres of nieuwsbrief)
- O Ander (*vul hieronder aan a.u.b.*):

…………………………………………………………………………………………..…………

7. Met welk informatiemiddel gebeurde dit? *(kruis **1** of **meerdere** bolletjes aan)*

- O powerpointpresentatie rond 10.000 Stappen
- O folder 10.000 Stappen
- O website van 10.000 Stappen
- O enkel mondelinge uitleg zonder bijkomend illustrerend materiaal
- O Ander (*vul hieronder aan a.u.b.*):

…………………………………………………………………………………………..…………

8. Duid aan in welke mate u het eens bent met volgende uitspraken *(kruis **1** bolletje per regel aan)*

	**helemaal mee oneens**	**mee oneens**	**niet oneens/ niet eens**	**mee eens**	**helemaal mee eens**
De inhoud en beschikbare instrumenten van '10.000 Stappen' zijn goed gekend door uw lokale organisatie.	O	O	O	O	O

	helemaal mee oneens	mee oneens	niet oneens/ niet eens	mee eens	helemaal mee eens
’10.000 Stappen’ betekent (kan) voor uw lokale organisatie een meerwaarde (betekenen) in vergelijking met andere, bestaande bewegingsprogramma’s/-projecten	o	o	o	o	o
’10.000 Stappen’ is complementair met de huidige doelstellingen en werking van uw lokale organisatie	o	o	o	o	o
’10.000 Stappen’ komt tegemoet aan noden van uw lokale organisatie	o	o	o	o	o
’10.000 Stappen’ is geschikt om dit eerst op experimentele basis (kleine schaal) te organiseren en nadien eventueel uit te breiden	o	o	o	o	o
’10.000 Stappen’ is complex om te begrijpen en te organiseren	o	o	o	o	o
’10.000 Stappen’ levert (kan) visibiliteit en zichtbare resultaten op (opleveren)	o	o	o	o	o
’10.000 Stappen’ is een geschikt product om zich als organisatie te kunnen profileren	o	o	o	o	o

Toepassing - algemeen

9. Heeft uw GVO-dienst of dienst gezondheidspromotie '10.000 Stappen' of een variant hiervan tot op heden zelf toegepast? (*kruis **1** bolletje aan*)

O Neen, en we hebben ook geen intentie om dit in de toekomst te doen. ***(→ga nu naar vraag 11)***

O Neen, en we weten nog niet of we dit zouden doen in de toekomst ***(→ga nu naar vraag 11)***

O Neen, maar we hebben wel de intentie om dit in de toekomst te doen. **(→*ga nu naar vraag 12*)**

O Ja, maar dit is reeds achter de rug ***(→ga nu naar vraag 10)***

O Ja, dit is nog gaande ***(→ga nu naar vraag 13)***

10. Hoe lang is het geleden dat uw '10.000 Stappen'-campagne of een variant hiervan is beëindigd? (*kruis **1** bolletje aan*)

O tot 1 maand geleden ***(→ga nu naar vraag 13)***

O 1 tot 3 maanden geleden ***(→ga nu naar vraag 13)***

O 4 tot 6 maanden geleden ***(→ga nu naar vraag 13)***

O 7 tot 12 maanden geleden ***(→ga nu naar vraag 13)***

O meer dan 12 maanden geleden ***(→ga nu naar vraag 13)***

11. Wat zijn de voornaamste redenen om '10.000 Stappen' of een variant hiervan niet toe te passen? *(kruis **max. 2** bolletjes aan)*

O we hebben hier nog niet concreet over nagedacht

O onvoldoende steun van directie of overste

O onvoldoende steun of motivatie onder de eigen collega's

O onvoldoende (geplande) financiële ruimte

O geen prioriteit/ niet geschikt voor onze lokale organisatie

O nood aan praktijkvoorbeelden en kennisondersteuning (bv. bijscholing)

O Ander (*vul hieronder aan a.u.b.*):

……………………………………………………………………………………..……………

→ Indien u zonet vraag 11 beantwoord heeft, eindigt voor u de vragenlijst hier. Dank voor uw medewerking!

12. Wat heeft het meest bijgedragen tot de beslissing om ‘10.000 Stappen’ of een variant hiervan in de toekomst toe te passen? *(kruis **max. 2** bolletjes aan)*

O de bewijzen uit de wetenschappelijke studie van het pilootproject in Gent
O de ervaringen van collega-organisaties uit een andere gemeente/regio
O opdracht van de directie of overste
O subsidies of andere ondersteuning
O 10 000 Stappen is een gebruiksklaar produkt
O Ander (*vul hieronder aan a.u.b*.):

…………………………………………………………………………………………..……………

→ Indien u zonet vraag 12 beantwoord heeft, eindigt voor u de vragenlijst hier. Dank voor uw medewerking!

13. Wat heeft het meest bijgedragen tot de beslissing om ‘10.000 Stappen’ of een variant hiervan toe te passen? *(kruis **max. 2** bolletjes aan)*

O de bewijzen uit de wetenschappelijke studie van het pilootproject in Gent
O de ervaringen van collega-organisaties uit een andere gemeente/regio
O opdracht van de directie of overste
O subsidies of andere ondersteuning
O 10 000 Stappen is een gebruiksklaar produkt
O Ander (*vul hieronder aan a.u.b*.):

…………………………………………………………………………………………..…………

14. Wat was/is de totale voorziene duur van uw ’10.000 Stappen’-campagne of variant hiervan (*kruis **1** bolletje aan*).

O 1 dag
O 2 dagen tot 1 week
O 2 weken tot 1 maand
O 2 tot 3 maanden
O 4 tot 6 maanden
O 7 tot 12 maanden
O Meer dan een jaar

15. Hoeveel personeelsleden werken/ werkten aan de '10.000 Stappen'-campagne of variant hiervan? (*kruis **1** bolletje aan*)

Gelieve hierbij geen rekening te houden met mogelijke inbreng van de landsbond.

- O 1 personeelslid
- O 2 personeelsleden
- O 3 personeelsleden
- O 4 personeelsleden
- O 5 of meer personeelsleden

16. Wanneer u alle werkdagen van de betrokken personeelsleden in acht neemt:

hoeveel werkdagen* (schatting) heeft uw ziekenfonds in het totaal aan de ‘10.000 Stappen’-campagne of variant hiervan besteed?

Indien uw campagne nog lopende is, gelieve dan ook het geschatte aantal werkdagen tot het einde van de campagne in het totaal te verrekenen!

(***vul in***) : +-………………………… **werkdagen**

* *Gelieve hierbij geen rekening te houden met mogelijke inbreng van de landsbond.*

17. Wat was/is voor uw ziekenfonds het totaal van de investering* (excl. personeelskosten) van uw ‘10.000 Stappen’-campagne of variant hiervan?

Indien uw campagne nog lopende is, gelieve dan ook de geschatte onkosten tot het einde van de campagne in het totaal te verrekenen.

(vul in)………………………………… **euro**

* *Gelieve hierbij geen rekening te houden met mogelijke inbreng van de landsbond.*

18. Op welke bewegingscontext(en) was/is uw ‘10.000 Stappen’-campagne of variant hiervan gericht? *(kruis **1** of **meerdere** bolletjes aan)*

- O beweging in de vrije tijd
- O actieve verplaatsing (te voet, per fiets naar de winkel, werk, de post...)
- O beweging op de werkvloer
- O beweging thuis

19. Tot welke leeftijdsgroep(en) was/is uw '10.000 Stappen'-campagne of variant hiervan gericht? *(kruis **1** of **meerdere** bolletjes aan)*

O kinderen en jongeren (<18 jaar)
O 18-tot-55-jarigen
O senioren (≥ 55 jaar)

20. Tot welke doelgroep(en) was/is uw '10.000 Stappen'-campagne of variant hiervan gericht? *(kruis **1** of **meerdere** bolletjes aan)*

O volledige bevolking van de gemeente/stad of regio
O het eigen personeel
O bedrijven
O scholen
O kansarmen
O Ander (*vul hieronder aan a.u.b.*):

……………………………………………………………………………………………………...

Toepassing - specifiek

21. Heeft u folders verspreid rond '10.000 Stappen' of een variant hiervan? (*kruis **1** bolletje aan*)

O Neen ***(→ga nu naar vraag 23)***
O Ja, op éénmalige basis ***(→ga nu naar vraag 22)***
O Ja, deze werden/worden meermaals verspreid of aangevuld ***(→ga nu naar vraag 22)***

22. Op welke schaal heeft u folders verspreid? (*kruis **1** bolletje aan*)

O Over het volledige werkgebied van mijn GVO-dienst of dienst gezondheidspromotie
O Beperkt tot volgende gemeenten of steden *(vul hieronder aan a.u.b):*

……………………………………………………………………………………………..

……………………………………………………………………………………………..

……………………………………………………………………………………………..

→ Indien u zonet vraag 22 beantwoord heeft, ga dan nu naar vraag 24.

23. Wat was/is de voornaamste reden om geen folders te verspreiden rond ’10.000 Stappen’ of een variant hiervan? *(kruis **max. 2** bolletjes aan)*

- O We overwegen deze mogelijkheid nog
- O Geen toestemming van de directie, overste of gemeente-/stadsbestuur
- O Te veel kosten
- O Dit behoort niet tot onze kerntaken
- O Nood aan meer inhoudelijke info/ondersteuning om deze folder op te stellen
- O Geen tijd
- O Ander *(vul hieronder aan a.u.b*.):

………………………………………………………………………………………………

→ Indien u zonet vraag 23 beantwoord heeft, ga dan nu naar vraag 25.

24. Waar heeft u deze folders verspreid? *(kruis **1** of **meerdere** bolletjes aan)*

- O Bedrijven
- O Bibliotheek
- O Gemeentehuis/openbare diensten van de gemeente
- O Plaatselijk kantoor van (het) ziekenfonds(en)
- O Scholen
- O Lokaal dienstencentrum of OCMW
- O Ander *(vul hieronder aan a.u.b*.):
- O Lokale handelaars of warenhuis
- O Praktijk van huisartsen, kinesisten en/of osteopaten
- O Praktijk van diëtisten
- O Gemeentelijke/stedelijke sporthal
- O Apothekers
- O Seniorenclub
- O Wijkvereniging of -centrum

…………………………………………………..

25. Heeft u gebruik gemaakt van baanaffiches[1] (of signalisaties) in het straatbeeld en/of gewone affiches? (*kruis **1** bolletje aan*)

- O Neen ***(→ga nu naar vraag 27)***
- O Ja, enkel baanaffiches of signalisaties in het straatbeeld ***(→ga nu naar vraag 26)***
- O Ja, enkel gewone affiches ***(→ga nu naar vraag 26)***
- O Ja, beide ***(→ga nu naar vraag 26)***

[1] Affiche voor op straat, langs wegen, bij parkings.

26. Op welke schaal heeft u baanaffiches (of signalisaties) in het straatbeeld en / of gewone affiches verspreid? (*kruis **1** bolletje aan*)

- O Over het volledige werkgebied van mijn GVO-dienst of dienst gezondheidspromotie
- O Beperkt tot volgende gemeenten of steden *(vul hieronder aan a.u.b):*

………………………………………………………………………………………………..

………………………………………………………………………………………………..

………………………………………………………………………………………………..

→ Indien u zonet vraag 26 beantwoord heeft, ga dan nu naar vraag 28.

27. Wat was/is de voornaamste reden om geen baanaffiches (of signalisaties) en/of gewone affiches te gebruiken? *(kruis **max. 2** bolletjes aan)*

- O We overwegen deze mogelijkheid nog
- O Geen toestemming van de directie, overste of gemeente-/stadsbestuur
- O Te veel kosten
- O Nood aan meer inhoudelijke info/ondersteuning om deze poster en baanaffiche op te maken
- O Dit behoort niet tot onze kerntaken
- O Geen tijd
- O Ander *(vul hieronder aan a.u.b.)*:

…………………………………………………………………………………………………

→ Indien u zonet vraag 27 beantwoord heeft, ga dan nu naar vraag 29.

28. Waar heeft u deze baanaffiches (of signalisaties) en/of gewone affiches opgehangen/geplaatst? *(kruis **1** of **meerdere** bolletjes aan)*

- O Bedrijven
- O Bibliotheek
- O Gemeentehuis/openbare diensten van de gemeente
- O Plaatselijk kantoor van (het) ziekenfonds(en)
- O Scholen
- O Lokale dienstencentrum of OCMW
- O Lokale handelaar of warenhuis
- O Parking van gemeentelijk (stads)centrum
- O Op wandelroutes
- O Op fietsroutes
- O Praktijk van huisartsen, kinesisten en/of osteopaten
- O Praktijk van diëtisten
- O Gemeentelijke/stedelijke sporthal
- O Apothekers
- O Seniorenclub
- O Wijkvereniging of -centrum
- O In lokale park
- O In lokale straten (gemeentelijk / stedelijk centrum)
- O Langs gemeentelijke / stedelijke autowegen
- O Ander *(vul hieronder aan a.u.b.)*:

…………………………………………………..

29. Heeft u gebruik gemaakt van interne of externe mediakanalen[2] om uw '10.000 Stappen'-campagne of een variant hiervan te promoten? (*kruis **1** bolletje aan*)

- O Neen ***(→ga nu naar vraag 31)***
- O Ja, op éénmalige basis ***(→ga nu naar vraag 30)***
- O Ja, deze werden/worden meermaals gebruikt ***(→ga nu naar vraag 30)***

30. Op welke schaal heeft u mediakanalen gebruikt? (*kruis **1** bolletje aan*)

- O Over het volledige werkgebied van mijn GVO-dienst of dienst gezondheidspromotie
- O Beperkt tot volgende gemeenten of steden *(vul hieronder aan a.u.b):*

………………………………………………………………………………………………..

………………………………………………………………………………………………..

………………………………………………………………………………………………..

→ Indien u zonet vraag 30 beantwoord heeft, ga dan nu naar vraag 32.

[2] Dit omvat het ruime spectrum aan mediakanalen: ledenbladen, kranten voor de inwoners (lokaal, regionaal, provinciaal of nationaal), al dan niet streekgebonden tijdschriften en magazines, regionale of nationale televisie

31. Wat was/is de voornaamste reden om geen mediakanalen te gebruiken? *(kruis **max. 2** bolletjes aan)*

- O We overwegen deze mogelijkheid nog
- O Geen toestemming van de directie, overste of gemeente-/stadsbestuur
- O Te veel kosten
- O Dit behoort niet tot onze kerntaken
- O Geen tijd
- O Ander *(vul hieronder aan a.u.b.)*:

…………………………………………………………………………………………….

→ Indien u zonet vraag 31 beantwoord heeft, ga dan nu naar vraag 33.

32. Welke mediakanalen heeft u aangewend? *(kruis **1** of **meerdere** bolletjes aan)*

- O Ledenblad
- O Gemeentelijk/stedelijk infoblad
- O De Streekkrant
- O Regionaal tijdschrift of magazine
- O Nationale krant (bv. Het Nieuwsblad)
- O Websites en/of e-zines
- O Vakspecifiek tijdschrift medische sector
- O Regionale televisie
- O Radio
- O Persconferentie/ persbericht
- O Ander *(vul hieronder aan a.u.b*.):

………………………………………………….

33. Heeft u pedometers of stappentellers ingeschakeld in functie van uw '10.000 Stappen'-campagne of variant hiervan? (*kruis **1** bolletje aan*)

- O Neen ***(→ga nu naar vraag 35)***
- O Ja ***(→ga naar vraag 34)***

34. Op welke schaal heeft u stappentellers ingeschakeld? (*kruis **1** bolletje aan*)

- O Over het volledige werkgebied van mijn GVO-dienst of dienst gezondheidspromotie
- O Beperkt tot volgende gemeenten of steden *(vul hieronder aan a.u.b):*

…………………………………………………………………………………………….

…………………………………………………………………………………………….

…………………………………………………………………………………………….

→ Indien u zonet vraag 34 beantwoord heeft, ga dan nu naar vraag 36.

35. Wat was/is de voornaamste reden om geen pedometers of stappentellers in te schakelen? *(kruis **max. 2** bolletjes aan)*

O We overwegen deze mogelijkheid nog
O Geen toestemming van de directie, overste of gemeente-/stadsbestuur
O Te veel kosten
O Dit behoort niet tot onze kerntaken
O Nood aan meer inhoudelijke info/ondersteuning om acties rond stappentellers op te zetten
O Geen tijd
O Ander: *(vul hieronder aan a.u.b.)*:

……………………………………………………………………………………………………

→ **Indien u zonet vraag 35 beantwoord heeft, ga dan nu naar vraag 40.**

36. Welk merk hebben de pedometers of stappentellers die zijn ingeschakeld / aangekocht? *(kruis **1** of **meerdere** bolletjes aan)*

O Yamax Digiwalker
O Omron - type 'Vital Steps' (HJ-005-E)
O Omron - type 'Active Steps' (HJ-109-E)
O Omron - type 'Walking Style I' (HJ-152-E)
O Omron - type 'Walking Style II' (HJ-113-E)
O Ander *(vul hieronder aan a.u.b.)*:

……………………………………………………………………………………………………

37. Hoe werden/worden de pedometers of stappentellers gebruikt/ aangeboden? *(kruis **1** of **meerdere** bolletjes aan)*

O gratis aanbod (gratis verdeling)
O voor individuele verkoop
O voor individuele verhuur tegen betaling
O voor individuele verhuur en gratis (al dan niet met waarborg)
O voor groepsverhuur (bv. werking met verhuurkoffers)
O Ander *(vul hieronder aan a.u.b.)*:

……………………………………………………………………………………………………

38. Werd/wordt er steeds een begeleidend (stappen)boekje[3] aan de koper/huurder aangeboden bij de pedometer of stappenteller? (*kruis **1** bolletje aan*)

**nooit**	**zelden**	**soms**	**vaak**	**altijd**
o	o	o	o	o

39. Waar werden de pedometers of stappentellers aangeboden? *(kruis **1** of **meerdere** bolletjes aan)*

- O In het gemeente-/stadshuis of openbare diensten van de gemeente/stad
- O Lokale dienstencentrum of OCMW
- O Plaatselijk kantoor / verkooppunt van (het) ziekenfonds(en)
- O Scholen
- O Bibliotheek
- O Praktijk van huisartsen, kinesisten en/of osteopaten
- O Apothekers
- O Seniorenclub
- O Wijkvereniging of –centrum
- O Ander *(vul hieronder aan a.u.b.*):

………………………………………………..

40. Heeft u samengewerkt met andere (gemeentelijke/stedelijke) diensten, instanties of verenigingen in functie van uw ‘10.000 Stappen’-campagne of variant hiervan? (*kruis **1** bolletje aan*)

- O Neen ***(→ga nu naar vraag 42)***
- O Ja ***(→ga nu naar vraag 41)***

41. Op welke schaal heeft u samengewerkt met andere (gemeentelijke/stedelijke) diensten, instanties of verenigingen? (*kruis **1** bolletje aan*)

- O Over het volledige werkgebied van mijn GVO-dienst of dienst gezondheidspromotie
- O Beperkt tot volgende gemeenten of steden *(vul hieronder aan a.u.b):*

……………………………………………………………………………………..

……………………………………………………………………………………..

……………………………………………………………………………………..

→ Indien u zonet vraag 41 beantwoord heeft, ga dan nu naar vraag 43.

---

[3] Hiermee bedoelen we een boekje dat het individu concreet begeleidt in de opbouw tot meer beweging in het dagelijks leven. Dit omvat aanbevelingen, tips en weekroosters waarin het individu zijn/haar weekdoelstelling en dagelijks stappenaantal kan neerschrijven gedurende 3 opeenvolgende maanden.

42. Wat was/is de voornaamste reden voor het feit dat er niet is/wordt samengewerkt met andere (gemeentelijke/stedelijke) diensten, instanties of verenigingen? *(kruis* ***max. 2*** *bolletjes aan)*

O We overwegen deze mogelijkheid nog
O De andere instantie(s) had(den)/heeft (hebben) geen interesse of de relatie is niet positief
O Geen toestemming van de directie, overste of gemeente-/stadsbestuur
O Dit biedt geen meerwaarde voor de campagne
O Dit behoort niet tot onze kerntaken
O Nood aan kennisondersteuning voor het opzetten van partnerships
O Geen tijd
O Ander *(vul hieronder aan a.u.b.)*:

…………………………………………………………………………………………………

→ Indien u zonet vraag 42 beantwoord heeft, ga dan nu naar vraag 45.

43. Met welke instanties/verenigingen heeft u samengewerkt in functie van het bereiken van de beoogde doelgroep(en)? *(kruis* ***1*** *of* ***meerdere*** *bolletjes aan)*

O Preventieadviseur, bedrijfsleider of medische dienst van bedrijf
O Bibliotheken
O Gemeente-/stadsbestuur
O Gemeentelijke/stedelijke sportdienst
O Gemeentelijke/stedelijke dienst mobiliteit
O Gemeentelijke/stedelijke dienst Welzijn of Gezondheid
O Lokaal dienstencentrum of OCMW
O Provinciale dienst (bv. sport, gezondheid, mobiliteit,…)
O Huisartsen, kinesisten en/of osteopaten
O Diëtisten
O (ander(e)) ziekenfonds(en)
O Apothekers
O Lokaal Gezondheidsoverleg (LOGO)
O Scholen
O Seniorenclub
O Wijkvereniging of -centrum
O Ander: *(vul hieronder aan a.u.b.)*:

………………………………………………………..

44. Is er in functie van uw '10.000 Stappen'-campagne of variant hiervan een stuurgroep of werkgroep gevormd met leden van de samenwerkende instanties/verenigingen? (*kruis* ***1*** *bolletje aan*)

O Neen
O Ja

45. Heeft u in functie van uw '10.000 Stappen'-campagne of variant hiervan de beoogde doelgroepen ook op een persoonlijke manier gecontacteerd of laten contacteren (bv. per mail, per brief, of telefonisch)? (*kruis **1** bolletje aan*)

O Neen ***(→ga nu naar vraag 47)***

O Ja, op éénmalige basis ***(→ga nu naar vraag 46)***

O Ja, meermaals ***(→ga nu naar vraag 46)***

46. Op welke schaal heeft u de beoogde doelgroepen op een persoonlijke manier gecontacteerd of laten contacteren? (*kruis **1** bolletje aan*)

O Over het volledige werkgebied van mijn GVO-dienst of dienst gezondheidspromotie

O Beperkt tot volgende gemeenten of steden *(vul hieronder aan a.u.b):*

…………………………………………………………………………………………..

…………………………………………………………………………………………..

…………………………………………………………………………………………..

→ Indien u zonet vraag 46 beantwoord heeft, ga dan nu naar vraag 48.

47. Wat was/is de voornaamste reden voor het feit dat u de beoogde doelgroepen niet op een persoonlijke manier gecontacteerd of laten contacteren heeft? *(kruis **max. 2** bolletjes aan)*

O We overwegen deze mogelijkheid nog

O Geen toestemming van de directie, overste of gemeente-/stadsbestuur

O Te veel kosten

O Dit behoort niet tot onze kerntaken

O Geen tijd

O Ander *(vul hieronder aan a.u.b.)*:

……………………………………………………………………………………………

48. Welke andere concrete acties werden/worden georganiseerd in functie van de '10.000 Stappen'-campagne of variant hiervan? *(kruis **1** of **meerdere** bolletjes aan)*

- O Interne acties voor het eigen personeel (bv. stappenwedstrijd, personeelsnamiddag,...)
- O Infostand of promo-acties rond '10.000 Stappen' gedurende publieke evenementen
- O Aangeven van afstanden tussen publieke plaatsen in aantal stappen (bv. van gemeente-/stadhuis tot station, …)
- O Uitstippelen van publieke (recreatieve) wandelparcours in aantal stappen
- O Themawandelingen in de gemeente met of zonder stappentellers (bv. bedrijfsincentives; natuurgebonden, toeristische, of culinaire themawandelingen,…).
- O Geen andere concrete acties
- O Andere *(vul hieronder aan a.u.b.)*:

……………………………………………………………………………………………

49. Op welke schaal werden andere concrete acties georganiseerd? (*kruis **1** bolletje aan*)

- O Over het volledige werkgebied van mijn GVO-dienst of dienst gezondheidspromotie
- O Beperkt tot volgende gemeenten of steden *(vul hieronder aan a.u.b):*

……………………………………………………………………………………………

……………………………………………………………………………………………

……………………………………………………………………………………………

50. Heeft u de website van '10.000 Stappen', met name www.10000stappen.be, al gebruikt? (*kruis **1** bolletje aan*)

- O Neen ***(→ga nu naar vraag 55)***
- O Ja ***(→ga nu naar vraag 51)***

51. Was u op de hoogte van de download-rubriek van de website waar u verschillende materialen (folder, affiches, begeleidende gids,...) kan downloaden in functie van een eigen '10.000 Stappen'-campagne of variant hiervan? (*kruis **1** bolletje aan*)

- O Neen ***(→ga nu naar vraag 54)***
- O Ja ***(→ga nu naar vraag 52)***

52. Geef aan of uw GVO-dienst (of dienst gezondheidspromotie) volgende materialen al gedownload en gebruikt heeft in functie van uw '10.000 Stappen'-campagne of afgeleide hiervan?

	Al ooit gedownload? (*kruis **1** bolletje aan per materiaal*)		Gebruikt voor de eigen '10.000 Stappen'-campagne of variant hiervan? (*kruis **1** bolletje aan per materiaal*)	
	**Neen**	**Ja**	**Neen**	**Ja**
Het 'stappen'-plan of implementatiegids voor beleidsinstanties en organisaties	○	○	○	○
Het 'stappen'-plan of implementatiegids voor bedrijven	○	○	○	○
Logo[4] of campagnebeeld[5] van 10.000 Stappen	○	○	○	○
Baanaffiche van 10.000 Stappen	○	○	○	○
Gewone affiche of poster van 10.000 Stappen	○	○	○	○

4 10000stappen.be

5 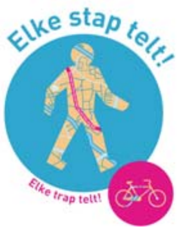

	Al ooit gedownload? (*kruis **1** bolletje aan per materiaal*)		Gebruikt voor de eigen '10.000 Stappen'-campagne of variant hiervan? (*kruis **1** bolletje aan per materiaal*)	
	**Neen**	**Ja**	**Ja**	**Neen**
Trapaffiche van 10.000 Stappen	O	O	O	O
Folder van 10.000 Stappen	O	O	O	O
Stappenboekje of registratieboekje van het pedometerpakket	O	O	O	O
Powerpointpresentatie	O	O	O	O

53. Duid aan voor elk van de downloadbare materialen in welke mate u deze nuttig vindt. (*kruis **1** bolletje aan per materiaal*)

Indien u een materiaal nog nooit gedownload heeft, kan u *'ken ik niet'* aanduiden.

	**Niet nuttig**	**Eerder niet nuttig**	**Soms niet/soms wel nuttig**	**Eerder wel nuttig**	**Wel nuttig**	***Ken ik niet***
Het 'stappen'-plan of implementatiegids voor bedrijven	O	O	O	O	O	O
Het 'stappen'-plan of implementatiegids voor beleidsinstanties en organisaties	O	O	O	O	O	O

	**Niet nuttig**	**Eerder niet nuttig**	**Soms niet/soms wel nuttig**	**Eerder wel nuttig**	**Wel nuttig**	***Ken ik niet***
Baanaffiche van 10.000 Stappen	○	○	○	○	○	○
Gewone affiche of poster van 10.000 Stappen	○	○	○	○	○	○
Trapaffiche van 10.000 Stappen	○	○	○	○	○	○
Folder van 10.000 Stappen	○	○	○	○	○	○
Logo[6] of campagnebeeld[7] van 10.000 Stappen	○	○	○	○	○	○
Stappenboekje of registratieboekje van het pedometerpakket	○	○	○	○	○	○
Powerpointpresentatie	○	○	○	○	○	○

6

7

54. Duid aan in welke mate u het eens bent met onderstaande algemene uitspraken met betrekking tot de website van ‘10.000 Stappen’. (*kruis **1** bolletje aan per uitspraak*)

	**helemaal mee oneens**	**mee oneens**	**niet oneens/ niet eens**	**mee eens**	**helemaal mee eens**
De verstrekte info op de website is geloofwaardig	O	O	O	O	O
De verstrekte info op de website is nuttig	O	O	O	O	O
U houdt van de globale presentatie van de website	O	O	O	O	O
U vindt makkelijk de info die u nodig heeft op de website	O	O	O	O	O
U vindt de rubrieken voor de afzonderlijke provincies een meerwaarde	O	O	O	O	O

55. Is er de intentie om in de toekomst een vervolgcampagne te plannen na de voorbije/ huidige ‘10.000 Stappen’-campagne of afgeleide hiervan? (*kruis **1** bolletje aan*)

O Neen ***(→ga naar vraag 56)***
O Ja, maar met een geringere investering **(voor u eindigt de vragenlijst hier)**
O Ja, en met eenzelfde grootte van investering **(voor u eindigt de vragenlijst hier)**
O Ja, en met een grotere investering **(voor u eindigt de vragenlijst hier)**
O Ja, maar we kennen de grootte van investering nog niet **(voor u eindigt de vragenlijst hier)**
O Dit weten we nog niet **(voor u eindigt de vragenlijst hier)**

56. Waarom zal er in de toekomst geen vervolgcampagne gepland worden? *(kruis **max. 2** bolletjes aan)*

O Te veel kosten
O Geen toestemming van de directie, overste of gemeente-/stadsbestuur
O Nood aan meer inhoudelijke info/ concrete producten ideeën voor vervolgacties
O Nood aan meer partners
O Dit behoort niet tot onze kerntaken
O Geen tijd
O De campagne had een teleurstellende respons bij de beoogde doelgroep(en)
O Ander *(vul hieronder aan a.u.b.)*:

………………………………………………………………………………………………………

Dank u wel voor uw medewerking!
